# Trends in sleep dentistry research in Asia: A bibliometric analysis

**DOI:** 10.12688/f1000research.164414.1

**Published:** 2025-05-12

**Authors:** Faizul Hasan, Mokh Sujarwadi, Lia Taurussia Yuliana, Ervina Restiwulan Winoto, An’nisaa Chusida, Hendrik Setia Budi

**Affiliations:** 1 Faculty of Nursing, Chulalongkorn University, Bangkok, Bangkok, Thailand; 2 Faculty of Nursing, Universitas Jember, Jember, East Java, Indonesia; 3 Department of Orthodontics, Faculty of Dental Medicine, Universitas Airlangga, Surabaya, East Java, Indonesia; 4 Department of Forensic Odontology, Faculty of Dental Medicine, Universitas Airlangga, Surabaya, East Java, Indonesia; 5 Department of Oral Biology, Dental Pharmacology, Universitas Airlangga, Surabaya, East Java, Indonesia

**Keywords:** Sleep Dentistry, Therapeutic Applications, Human Wellbeing, Collaborative Research, Health Equity in Asia

## Abstract

**Background:**

Sleep-related conditions such as obstructive sleep apnea and bruxism significantly affect both oral and systemic health, posing substantial public health challenges. Growing scholarly interest in sleep dentistry reflects an emerging effort to address these conditions through multidisciplinary research. This study employs bibliometric analysis to examine emerging themes, collaborative patterns, influential authors, and research trajectories related to sleep dentistry in Asia.

**Methods:**

A comprehensive search was conducted using the Scopus database to identify relevant publications from inception through April 2025. Bibliometric techniques were applied to analyze co-authorship networks, annual publication trends, institutional and international collaborations, keyword co-occurrence, and citation metrics. VOSviewer and the Bibliometrix package in R were utilized for data visualization and network mapping.

**Results:**

The analysis included 1,237 publications. China was the leading contributor, followed by the United States and India. The United Kingdom exhibited the highest ratio of Multiple Country Publications, followed by Australia and Canada. Tehran University of Medical Sciences emerged as the most productive institution, followed by the All India Institute of Medical Sciences and Shahid Beheshti University of Medical Sciences. Co-authorship analysis revealed six distinct collaborative clusters, with a total of 5,828 scholars contributing to the field.

**Conclusion:**

A substantial and growing body of research on sleep dentistry has emerged in Asia. The bibliometric findings highlight influential contributors, international cooperation, and key research themes particularly obstructive sleep apnea and bruxism underscoring the value of bibliometric methods in shaping responses to this pressing regional public health concern.

## Introduction

Sleep dentistry, or dental sleep medicine, is a burgeoning interdisciplinary domain that emphasizes the diagnosis and treatment of sleep-related problems, including obstructive sleep apnea (OSA) and other sleep-breathing complications. This discipline has gained prominence as dentists increasingly acknowledge their essential role in detecting these conditions through routine examinations and patient interactions, which can disclose symptoms such as snoring and daytime hypersomnolence ( Correa & Acosta-Torres, 2024; Smith & Smith, 2017). The amalgamation of dental procedures with sleep medicine facilitates unique treatment alternatives, including the utilization of bespoke intraoral gadgets that are both efficacious and economical ( de Oliveira, de Oliveira Júnior, & Guedes, 2023; Mazumder). Moreover, the collaboration of dentists, sleep specialists, and other healthcare practitioners improves patient care by addressing the intricate relationship between dental health and sleep quality ( Cistulli & Balasubramaniam, 2024; Lobbezoo et al., 2020). With the advancement of research and clinical practices, dental sleep medicine is anticipated to emerge as a recognized specialty, markedly enhancing the management of sleep disorders and overall patient well-being.

Recent studies in sleep dentistry in Asia underscore notable developments and trends that conform to worldwide norms while also tackling region-specific challenges. There has been a notable increase in interest in the topic, especially regarding OSA, which is a significant concern due to its substantial effects on quality of life and related health risks, including cardiovascular disorders ( Wadhawan et al., 2024). The formation of entities such as the Asian Sleep Research Society (ASRS) and the Asian Society of Sleep Medicine (ASSM) has promoted the advancement of sleep research and education in 29 Asian nations, highlighting deficiencies in accreditation and training that require rectification ( BaHammam et al., 2024; Okuma, 1995). Moreover, multidisciplinary collaboration among dentists, sleep physicians, and other healthcare providers is increasingly prioritized, mirroring a global trend towards integrated care in the management of sleep-related diseases ( Correa & Acosta-Torres, 2024). Asia's study on sleep dentistry reflects global trends while distinctly emphasizing cultural and epidemiological factors relevant to the region ( Okuma, 1995; Wadhawan et al., 2024).

Despite sleep dentistry is increasingly acknowledged as a vital interdisciplinary discipline that addresses sleep-related respiratory issues and their implications for dental health, research in this area remains comparatively underdeveloped in most Asian nations. Despite the heightened global interest in sleep dentistry, significant discrepancies remain in research output, theme concentration, methodological robustness, and international collaboration within the Asian area ( Herrero Babiloni & Lavigne, 2018; Huang, 2009; Niu et al., 2023; Pan et al., 2022). Furthermore, the integration of emerging technologies—such as digital diagnostics, telemedicine, and machine learning—continues to be constrained within the regional context ( Hasan et al., 2024; Joda et al., 2020; Perez-Pozuelo et al., 2020; Van Noort, 2012). Structural and structural obstacles, such as inadequate research funding, restricted access to modern diagnostic technology, a deficiency of trained personnel, and disjointed policy frameworks, further hinder advancement in this domain ( BaHammam et al., 2024; BaHammam et al., 2025; Cherasse, 2011). These constraints impede the advancement of substantial, context-specific evidence and diminish the prominence of Asian research in global discussions and guideline creation. To rectify this deficiency, it is essential to carefully delineate the landscape of sleep dentistry research in Asia utilizing bibliometric analysis.

Bibliometric analysis holds limited importance, as it only slightly enhances researchers' comprehension of the topic and offers ambiguous indications for possible avenues of future exploration. This methodology enables the discernment of noteworthy trends, influential authors, collaborative networks, and thematic gaps, thereby offering valuable insights for tackling regional challenges to enhance local research capabilities and foster global integration. The results obtained from our study reveal important insights and suggest novel avenues for further investigation within this swiftly evolving domain of knowledge and research exploration.

## Methods

### Search strategy

We performed a search for relevant articles in the Scopus database, acknowledged as one of the largest global archives of academic literature. Scopus includes around 24,000 contemporary publications from over 5,000 international publishers, comprising peer-reviewed journals, conference proceedings, commercial magazines, and academic book series. The database has around 75 million records, encompassing diverse types of intellectual outputs including journals, conference proceedings, patents, and publications across numerous disciplines. Our literature search included the following keywords: (ALL (sleep) AND ALL (dentistry) AND ALL (Asia)). Two experts with at least five years of experience in systematic reviews validated our search method following a thorough evaluation of all article titles collected. They identified all relevant publications, resolving any differences by discussion with an extra author. All retrieved articles were ultimately retained.

### Screening

Only articles that employed their findings and concentrated on sleep and dentistry were included in the bibliometric analysis. No language restrictions were imposed. Letters, editorials, conference abstracts, and book chapters were excluded from the analysis, whereas research papers and reviews published in peer-reviewed journals were included. The assessment of the papers was performed by two experts in bibliometric analysis, who had considerable expertise and evaluated the publications based on established criteria and recommendations ( Martinez, Al-Hussein, & Ahmad, 2019).

### Bibliometric analysis

To generate replicable and quantifiable data relevant to policy administration, bibliometric analysis ( Pritchard, 1969) functions as a quantitative evaluation of scientific research, scrutinizing contemporary research trends within a designated field ( Mooghali et al., 2011). Bibliometric analysis possesses the potential to provide an extensive exploration of a particular area of inquiry, while also identifying research fields that academics should explore and the strategies that authors have devised to fulfill their aims ( Su & Lee, 2010). A comprehensive portrayal of the field enables readers to more effectively grasp the patterns and trends present in sleep dentistry research.

Furthermore, the distribution, proportions, and frequency for each journal were delineated. The classifications, ratios, and frequencies for each publication were displayed. The relevant author ratios, frequency, and percentage were calculated for each country, both individually and collectively. The frequency and citation rate for each author and organization were also presented. The research influence of each nation, journal, organization, and author was organized according to the number of publications. All data was generated and illustrated using VOSviewer (Leiden University) and Bibliometrix (an R package).

## Results

### Publication output

The concluding bibliometric analysis comprised a total of 1,237 papers following the screening procedure.

**Figure 1.**
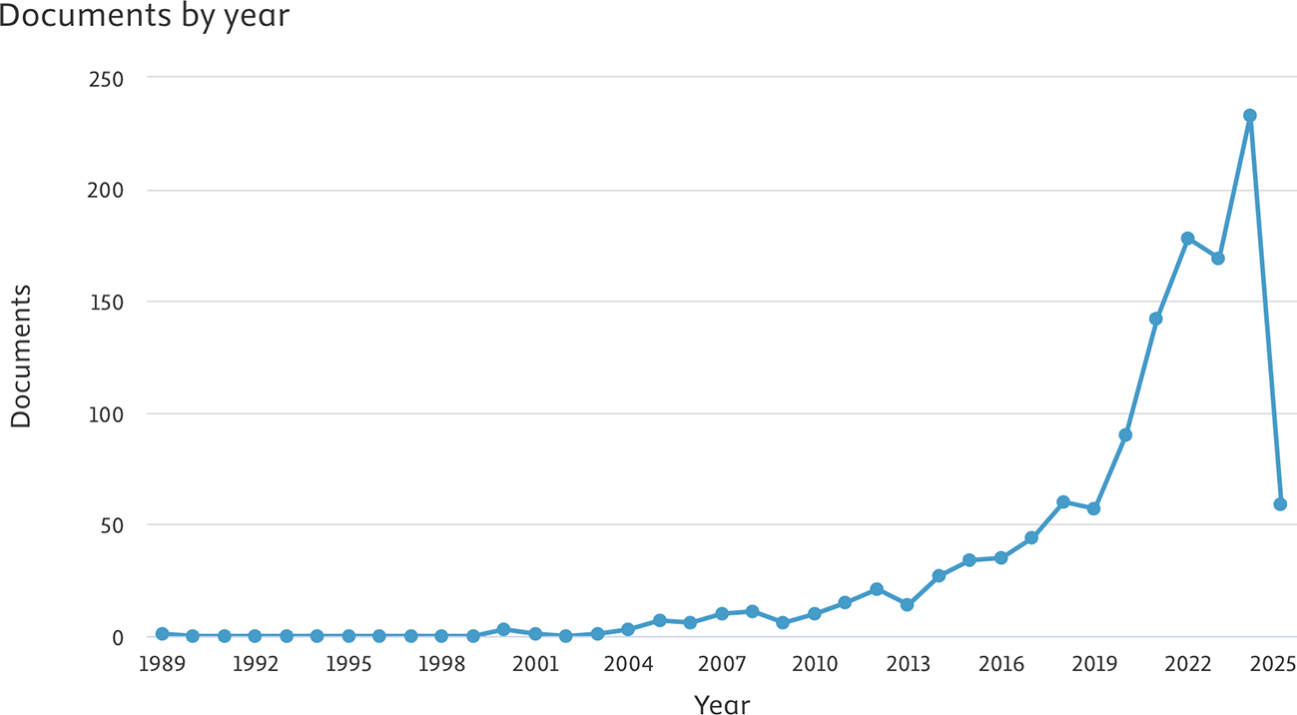
The distribution of articles by year.

Figure 1 illustrates the increase in the volume of articles pertaining to sleep dentistry. Table 1 illustrates the geographical distribution of the top ten nations engaged in research pertaining to sleep dentistry. China distinguished itself as the foremost contributor, presenting 130 publications, followed by the United States of America with 96 publications, and India with 61 publications. The nation exhibiting the preeminent ratio of Multiple Country Publications (MCP) was the United Kingdom, with a figure of 62.7, succeeded by Australia at 60.3 and Canada at 58.5.

**Table 1.** Top 10 countries that published in 1989–2025.

Rank	Country	Articles	Articles %	SCP	MCP	MCP %
1	China	130	11.2	81	49	37.7
2	Usa	96	8.3	50	46	47.9
3	India	61	5.3	41	20	32.8
4	United Kingdom	59	5.1	22	37	62.7
5	Australia	58	5	23	35	60.3
6	Canada	53	4.6	22	31	58.5
7	Japan	43	3.7	32	11	25.6
8	Iran	30	2.6	23	7	23.3
9	Brazil	29	2.5	20	9	31
10	Malaysia	28	2.4	17	11	39.3

MCP= multiple country publications; SCP= single country publications.

**Figure 2.**
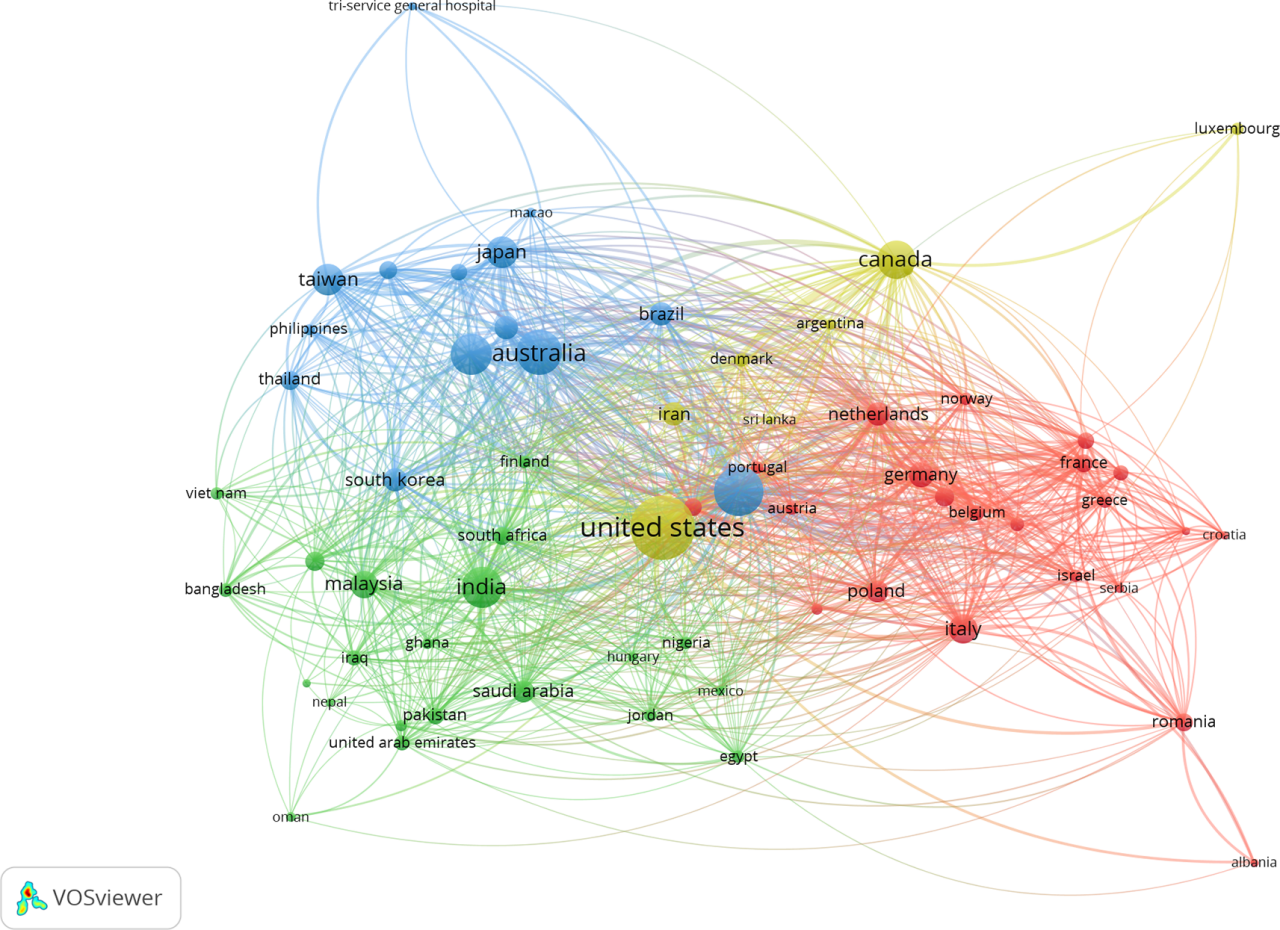
The co-authorship network of country that contributed to the topic during 1989 and 2025.

Figure 2 illustrates the interconnected web of nations that have engaged in a minimum of five collaborative research endeavors.

Supplementary Table 1 presents the top ten institutions that have demonstrated significant productivity in the realm of sleep dentistry research. Tehran University of Medical Sciences emerges as the most distinguished institution, boasting an impressive total of 336 published articles. It is followed by the All India Institute of Medical Sciences, which has contributed 192 publications, and the Shahid Beheshti University of Medical Sciences, with a commendable 167 articles to its name.

The VOSviewer tool was utilized to conduct a co-authorship analysis, with the objective of providing a comprehensive representation of the complete network of countries engaged in sleep dentistry research. The extent of collaboration between two nations is ascertained by the number of publications they have jointly authored, with each nation contributing at least five articles to the collective output. The analysis of authorship by country revealed the emergence of six distinct clusters, as depicted in Supplementary Figure 1.

A total of 5,828 experts contributed to the publication of 1,237 scientific articles on sleep dentistry research. Supplementary Table 2 provides a comprehensive overview of the ten most prolific authors in the field of sleep dentistry research. Wang Y is a notable author with 36 scholarly works on the subject. The author's impact is also detailed in Supplementary Table 3.

### Co-occurrence analysis of top 10 keywords

**Figure 3.**
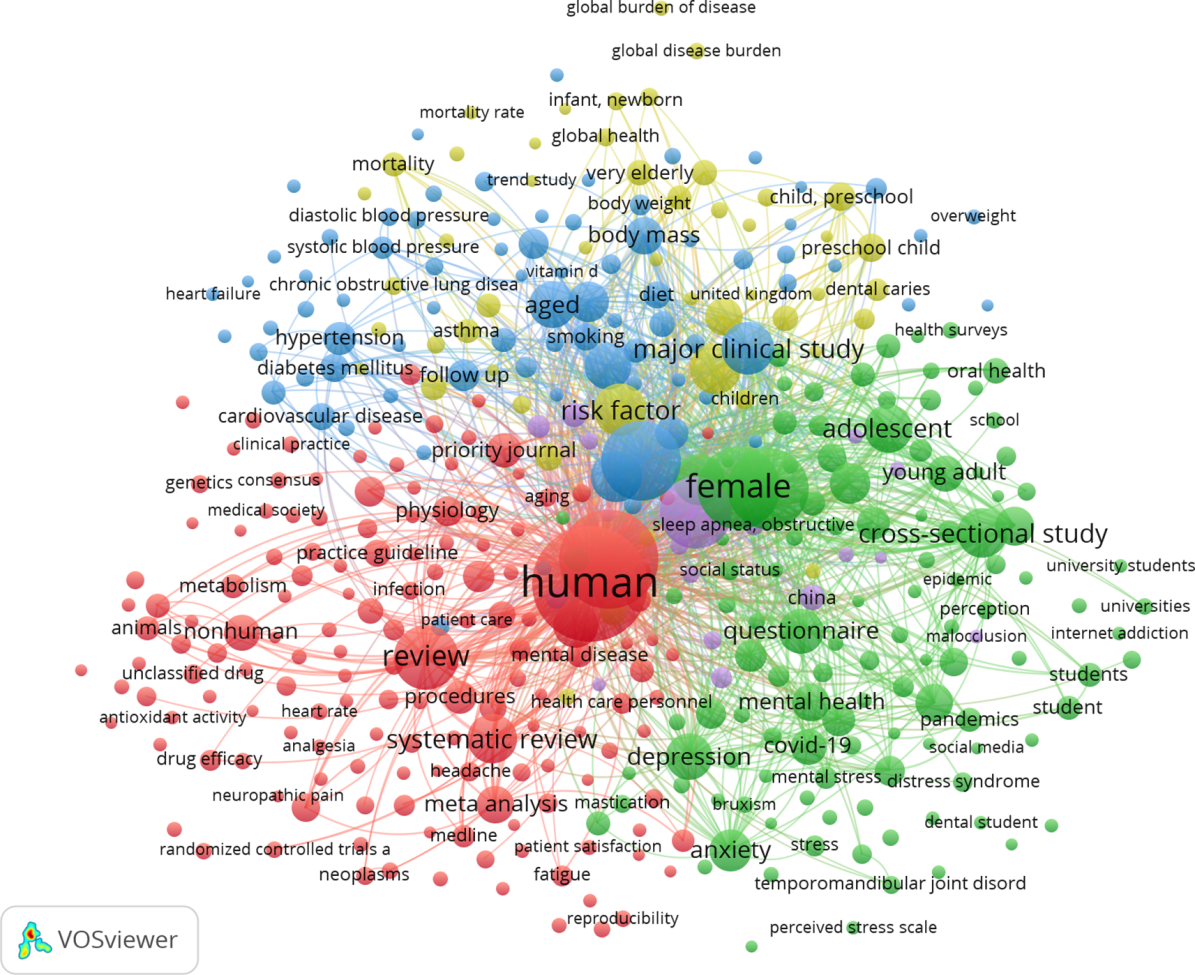
The co-occurrence network of the top 10 keywords in this research, 1989–2025. The top 10 keywords were collected and clustered using the visualization application VOSviewer. We used VOSviewer to create a visual network map of the top 10 keywords in five clusters, including their co-occurrence. The colors denote the 5 clusters: red (cluster 1, 153 items), green (cluster 2, 116 items), blue (cluster 3, 79 items), yellow (cluster 4, 49 items), and purple (cluster 5, 18 items). The node label is the keyword, and the number of keywords determines the size of the label. The connection joins two nodes that represent the relationship of two terms.

The 1,237 articles were categorized into five keyword clusters. The 10 most often used terms in the retrieved articles are listed in Supplementary Table 4. The analysis of the co-occurrence of the top 10 terms identified the research hotspots in the domain of dental caries ( Figure 3).

### Reference co-citation analysis

We explored the knowledge bank of the sleep dentistry research field in greater depth. Table 2 presents the ten papers with the highest citation counts. Livingston G, 2020, LANCET, achieved the highest citation count by April 12, 2025, with a total of 6,465 citations. This article was published in The Lancet in 2020.

**Table 2.** Top 10 high cited articles 1989–2025.

Rank	First author/Journal	DOI	Total citations	TC per year
1	Livingston G, 2020, Lancet	10.1016/S0140-6736(20)30367-6	6465	1,077.50
2	Klionsky DJ, 2016, Autophagy	10.1080/15548627.2015.1100356	4453	445.30
3	Shilling C, 2012, The Body and Soc Theory	10.4135/9781473914810	2771	197.93
4	Kyu Hh, 2018, Lancet	10.1016/S0140-6736(18)32335-3	2386	298.25
5	Forouzanfar Mh, 2015, Lancet	10.1016/S0140-6736(15)00128-2	2272	206.55
6	Umemura S, 2019, Hypertens Res	10.1038/s41440-019-0284-9	1309	187.00
7	Shimamoto K, 2014, Hypertens Res	10.1038/hr.2014.20	938	78.17
8	Bouayed J, 2010, Oxid Med Cell Longev	10.4161/oxim.3.4.12858	890	55.63
9	Ogihara T, 2009, Hypertens Res	10.1038/hr.2008.18	882	51.88
10	Gallagher Km, 2012, Ann Behav Med	10.1007/s12160-011-9308-7	828	59.14

DOI= digital object identifiers; TC= total citation.

## Discussion

This study represents, to our understanding, the inaugural bibliometric analysis concentrated specifically on the domain of sleep dentistry. The findings suggest an increasing engagement from Asian scholars in investigating novel methodologies and utilizing cutting-edge technologies to tackle significant challenges within this field. The consistent rise in publications from 1989 to 2025 emphasizes the growing significance of sleep dentistry within both academic and clinical spheres, reflecting its developing role as a pertinent public health issue in the region.

Research on sleep dentistry in Asia has garnered significant attention recently, with China emerging as a primary contributor, followed by the United States and India. These countries have emerged as essential contributors to the advancement of the discipline and the enhancement of academic production. Research undertaken throughout the region has enhanced comprehension of sleep-associated dental disorders—specifically OSA and bruxism—and their ramifications for both oral and systemic health ( Tay et al., 2020; Wan Yong Tan et al., 2019; Yap et al., 2023). By integrating several data sources, such as craniofacial morphology, polysomnographic evaluations, behavioral elements, and demographic attributes, researchers have identified essential risk variables and improved diagnostic and therapeutic approaches ( Flores-Mir et al., 2013). These patterns indicate a rising worldwide and regional interest in sleep dentistry, with Asian nations assuming an increasingly significant role in the field's advancement ( BaHammam et al., 2024; Neelapu et al., 2017; Wan, 2024). Collaborative efforts among academics, physicians, and academic institutions around Asia highlight the region's dedication to enhancing sleep-related oral health studies and increasing clinical outcomes ( Lyons et al., 2020).

The research environment of sleep dentistry in Asia demonstrates significant productivity among prominent universities. Tehran University of Medical Sciences is the foremost university in this domain, demonstrating a robust dedication to furthering research on sleep-related oral disorders, specifically obstructive sleep apnea and bruxism. The All India Institute of Medical Sciences actively engages in the scholarly discussion regarding the impact of sleep dentistry on enhancing dental and systemic health. Shahid Beheshti University of Medical Sciences holds the third position, thereby enhancing the global corpus of knowledge regarding sleep dentistry and its therapeutic applications. The commitment of these universities to sleep dentistry research highlights a regional initiative in Asia aimed at improving diagnostic precision and treatment results in dental healthcare for patients with sleep-related illnesses.

Of note, the utilization of pharmacological agents, especially drugs and hypnotics, in the treatment of OSA has received considerable focus in recent years. Although continuous positive airway pressure (CPAP) therapy is the established gold standard for the treatment of OSA, pharmaceutical methods are being investigated as supplementary or alternative therapies, particularly for individuals who are intolerant to CPAP or want non-invasive solutions ( Bosi et al., 2021; Khurana et al., 2021). Hypnotic drugs, including benzodiazepines and non-benzodiazepine sleep aids, are occasionally employed to enhance sleep quality and diminish arousals linked to OSA ( Carter & Eckert, 2021; Hasan et al., 2023; Sys et al., 2020); nonetheless, their administration necessitates vigilant oversight due to the risk of respiratory depression and exacerbation of airway blockage ( Tietjens et al., 2019). Alternative medications, including modafinil, have been examined for their efficacy in reducing daytime somnolence in people with OSA, however they do not rectify the fundamental airway obstruction ( Kuan et al., 2016; Pitre et al., 2023). The utilization of innovative pharmacotherapies, such as serotonin agonists and orexin receptor antagonists, demonstrates potential in modulating sleep-wake cycles and enhancing sleep architecture and respiratory performance during sleep ( Clark et al., 2020; Janto, Prichard, & Pusalavidyasagar, 2018; Kron et al., 2024). Nonetheless, these pharmacological interventions include dangers, necessitating meticulous patient selection and monitoring in the management of OSA to reconcile therapeutic advantages with safety issues ( Horner, 2023).

Although this bibliometric analysis offers intriguing insights, it is essential to acknowledge the significant limitations that must be considered. The reliance on the Scopus database could lead to the exclusion of important articles from diverse sources. The accuracy of the metadata linked to the articles further constrains the study. Furthermore, bibliometric analysis emphasizes quantitative assessments over qualitative evaluations of research outcomes and methodologies. In light of these constraints, the findings underscore the growing significance of exploring the overarching trends in sleep dentistry across Asia, which could inform future therapeutic approaches and research directions.

## Conclusion

We have observed a growing body of research in the field of sleep dentistry throughout Asia. The bibliometric analysis delineates prominent authors, collaborative networks, and nascent topics, encompassing obstructive sleep apnea, bruxism, and their interplay with dental health. This observation highlights an emerging trend in research spanning from 1989 to 2025. This study underscores the significance of bibliometric analysis in tackling this pressing public health concern within the region.

## Reporting guidelines

Zenodo: strobe checklist sleep dentistry, https://doi.org/10.5281/zenodo.15205065 ( Hasan, 2025b).

Data are available under the terms of the Creative Commons Attribution 4.0 International license (CC-BY 4.0).

**Software**: VOSviewer is a free software tool for constructing and visualizing bibliometric networks. To learn more, visit the VosViewer Getting Started page.

## Ethics and consent

Ethical approval and consent were not required.

## Data Availability

No data are associated with this article. Supplementary materials are available in the supplementary data. Zenodo: supplementary files sleep dentistry, https://doi.org/10.5281/zenodo.15205061 ( Hasan, 2025a). This project contains the following underlying data: supplementary files.zip Data are available under the terms of the Creative Commons Attribution 4.0 International license (CC-BY 4.0).
