# Supplementary material for: Trends in sleep dentistry research in Asia: A bibliometric analysis
Source: F1000Res. 2025 Aug 21;14:489. Originally published 2025 May 12. [Version 2] doi: 10.12688/f1000research.164414.2 (PMC12355164; doi:10.12688/f1000research.164414.2)
Supplement: Peer review history

# Reviewer report: Reviewer response for version 2

**DOI:** 10.5256/f1000research.186772.r405026

**Published:** 2025-09-05

**Authors:** Zahreni Hamzah, Didin Erma Indahyani

**Affiliations:** 1 Oral Biology, Universitas Jember (Ringgold ID: 175448), Jember, East Java, Indonesia; 2 University of Jember, Jember Regency, Indonesia; 3 University of Jember, Jember, Indonesia

ABSTRACT REVISION

1. This revised version of ABSTRACT significantly improves the quality of the original study through methodological refinements and a sharper conceptual focus. Search strategies now use comprehensive Boolean queries with *field-specific tags* to ensure stronger literature coverage. This analysis more clearly shows the value of bibliometrics in mapping research gaps and guiding evidence-based policy decision-making in the field of *sleep dentistry* in Asia. Important additions include practical recommendations for regional collaboration, funding allocation, and clinical practice improvement, particularly in addressing gaps such as sleep disorders in children. This revision strengthens the rigour of the research while increasing its translational impact for researchers, policymakers, and clinicians seeking to advance sleep medicine in Asia.
2. It is necessary to include the results of data normalization, because the publications produced differ from each discipline, country and institution, so a more relevant and comparatively valid picture of data is obtained. In addition, it will support the analysis of the disciplinary and institutional context of the article's origin.

INTRODUCTION REVISION, For this manuscript to be of higher quality, it would be better if:

1.  
*Sleep Dentistry* Research. It is very interesting and could be considered for indexing, as it is interdisciplinary and innovative, linking the field of dentistry with the fields of neurology, cardiology and pulmonology.
2. With the strengthening of methodologies, technological innovation, and regional collaboration, sleep dentistry has the potential to develop into an official speciality in dentistry.
3. It is better to add a more explicit research gap/research objective.
4. Provide the latest data/literature to be more evidence-based, especially in dentistry, so it seems to be an urgent matter, for example, how many cases, what has been done and how the results are, etc.
5. Create a more concise grouping of weaknesses & prospects; if possible, create a concise table.
6. Close each paragraph with its key message/urgency to be more argumentative, not just descriptive.
7. In the second paragraph, it is written "de Oliveira, de Oliveira Júnior, & Guedes, 2023; Mazumder .. What does this mean?

METHOD REVIEW, Suggestions for improvement:

1. The method is quite representative, but it is necessary to use international *bibliometric review* standards, using transparency aspects (PRISMA). Include a PRISMA flowchart.
2. Include the search period and the date of the last search.
3. It is necessary to explain the type of bibliometric analysis (e.g. co-citation, keyword analysis, bibliographic coupling).

REVIEW RESULT

 The analysis results are quite representative descriptively, but for the quality of indexing in international journals, it is necessary to add citation analysis (impact-based), not only the number of publications in the Asian regional context.

 REVIEW DISCUSSION

1. An extensive and multidimensional literature has supported the discussion. For the article to improve, the discussion structure needs to be more systematic, and the presentation of the research gap needs to be sharper.
2. The limitations of the Scopus database have been discussed, but a detailed discussion of them, such as their origin, potential impact and interpretation, is lacking, and gives concrete advice on the findings.
3. Attributing conformity or difference to previous research and its potential implications is necessary.
4. It is recommended that the implications of findings be reviewed in the context of clinical practice, policy, or research development.

REVIEW CONCLUSION

1. Your conclusions are already solid, but they need to be condensed, made more systematic and concise, followed by a number of supporting publications and explicit recommendations on the implications of the findings.
2. It is better to emphasize the added value of research and pave the way for further study.

Is the work clearly and accurately presented and does it cite the current literature?

Yes

If applicable, is the statistical analysis and its interpretation appropriate?

Partly

Are all the source data underlying the results available to ensure full reproducibility?

Partly

Is the study design appropriate and is the work technically sound?

Yes

Are the conclusions drawn adequately supported by the results?

Yes

Are sufficient details of methods and analysis provided to allow replication by others?

Partly

Reviewer Expertise:

Physio-Geriatry Oromaxillofacial

We confirm that we have read this submission and believe that we have an appropriate level of expertise to confirm that it is of an acceptable scientific standard, however we have significant reservations, as outlined above.

# Reviewer report: Reviewer response for version 1

**DOI:** 10.5256/f1000research.180922.r405030

**Published:** 2025-08-14

**Authors:** Yuni Asri

**Affiliations:** 1 Institut Teknologi, Sains dan Kesehatan RS dr Soepraoen Kesdam V/Brawijaya, Malang, Indonesia

The present work offers a thorough bibliometric analysis of research trends in sleep dentistry throughout Asia, utilizing a meticulous technique to chart the field's evolution from 1989 to 2025. The research adeptly employs Scopus data and sophisticated bibliometric instruments to elucidate significant trends in publication output, global collaborations, and research focal points, particularly with obstructive sleep apnea and bruxism. The identification of main contributors (China, USA, India) and premier universities offers significant benchmarks for comprehending the current research landscape in this nascent topic.

 The analysis is comprehensive; nonetheless, the study might be improved by more explicitly addressing specific limitations, such as potential database biases and language constraints that may influence the results. Incorporating sub-regional comparisons within Asia and examining the influence of healthcare infrastructure disparities on research output would provide essential background. The conclusion might be enhanced by offering more precise recommendations for future research avenues, especially with technological applications and comparative analyses across various healthcare systems.

 This research significantly contributes to dental sleep medicine by thoroughly documenting the development and current status of sleep dentistry research in Asia. The findings provide significant insights for researchers, clinicians, and policymakers aiming to enhance both academic knowledge and clinical practice in this vital field of healthcare. The study's rigorous approach and lucid presentation render it a significant reference for future endeavors in this field.

Is the work clearly and accurately presented and does it cite the current literature?

Yes

If applicable, is the statistical analysis and its interpretation appropriate?

Yes

Are all the source data underlying the results available to ensure full reproducibility?

Yes

Is the study design appropriate and is the work technically sound?

Yes

Are the conclusions drawn adequately supported by the results?

Yes

Are sufficient details of methods and analysis provided to allow replication by others?

Yes

Reviewer Expertise:

public health, community nursing, epidemiology

I confirm that I have read this submission and believe that I have an appropriate level of expertise to confirm that it is of an acceptable scientific standard.

# Reviewer report: Reviewer response for version 1

**DOI:** 10.5256/f1000research.180922.r384726

**Published:** 2025-06-03

**Authors:** Noengki Prameswari

**Affiliations:** 1 Universitas Hang Tuah, Surabaya, Indonesia

1. Introduction : Clarify your stance and strengthen the justification for why bibliometric analysis is appropriate and necessary for this research context. The potential contributions of the study are implied but not explicitly stated in a strong concluding sentence. The article should emphasize what novel insight this bibliometric review offers that prior literature has not.
2. Method : The search string (ALL (sleep) AND ALL (dentistry) AND ALL (Asia)) is simplistic and may miss relevant studies. It’s also not clear whether this refers to title, abstract, or full-text search. Provide the full Boolean search query as used in Scopus, including field tags (e.g., TITLE-ABS-KEY), and specify whether synonyms or variations were considered (e.g., "oral health," "OSA," "sleep apnea," etc.).
3. Discussion : While the findings are described, their broader implications for **policy, collaboration, or clinical practice** in Asia are only lightly touched upon. Add a paragraph that synthesizes how these results could inform national research agendas, promote regional networks, or guide funding priorities.
4. Conclusion : The final sentence, *"underscores the significance of bibliometric analysis in tackling this pressing public health concern,"* is somewhat vague. Be more specific—what exactly does bibliometric analysis contribute (e.g., strategic direction, research gap identification)?

Is the work clearly and accurately presented and does it cite the current literature?

Yes

If applicable, is the statistical analysis and its interpretation appropriate?

Yes

Are all the source data underlying the results available to ensure full reproducibility?

Yes

Is the study design appropriate and is the work technically sound?

Yes

Are the conclusions drawn adequately supported by the results?

Partly

Are sufficient details of methods and analysis provided to allow replication by others?

Yes

Reviewer Expertise:

Orthodontic, Physiology

I confirm that I have read this submission and believe that I have an appropriate level of expertise to confirm that it is of an acceptable scientific standard.
